# Performance enhancement of polysiloxane-based nanocomposite TENGs through optimized MWCNT concentration

**DOI:** 10.3389/fchem.2026.1689849

**Published:** 2026-03-04

**Authors:** Talia Tene, Orkhan Gulahmadov, Lala Gahramanli, Mustafa Muradov, Nahida Musayeva, Stefano Bellucci, Christos Trapalis, Gabriela Tubon-Usca, Carlos Ramiro Peñafiel-Ojeda, Cristian Vacacela Gomez

**Affiliations:** 1 Department of Chemistry, Universidad Técnica Particular de Loja, Loja, Ecuador; 2 Nano Research Laboratory, Center of Excellence, Baku State University, Baku, Azerbaijan; 3 Faculty of Engineering, Karabakh University, Khankendi, Azerbaijan; 4 Institute of Physics, Ministry of Science and Education of Azerbaijan, Baku, Azerbaijan; 5 Department of Application Technologies of Nanomaterials, National Institute of Materials Physics, Magurele, Romania; 6 Institute of Nanoscience and Nanotechnology, National Center for Scientific Research “Demokritos”, Agia Paraskevi, Greece; 7 Facultad de Ciencias, Escuela Superior Politécnica de Chimborazo (ESPOCH), Riobamba, Ecuador; 8 Universidad Nacional de Chimborazo. Facultad de Ingeniería. Carrera de Telecomunicaciones, Grupo de Investigación en Telecomunicaciones, Informática, Industria y Construcción (TEIIC), Riobamba, Ecuador; 9 Department of Environmental Engineering (DIAm), University of Calabria, Rende, Italy; 10 Universidad Ecotec, Samborondon, Ecuador

**Keywords:** dielectric materials, MWCNTs, nanocomposite film, nylon, polysiloxane, TENG

## Abstract

**Introduction:**

This study examines the effect of multi-walled carbon nanotube (MWCNT) loading on the dielectric behavior and triboelectric performance of polysiloxane (PS)-based nanocomposites for high-efficiency triboelectric nanogenerators (TENGs).

**Methods:**

Flexible PS/MWCNT films were fabricated using the doctor blading method and characterized by Raman spectroscopy and scanning electron microscopy (SEM). Broadband dielectric spectroscopy was employed to analyze frequency-dependent permittivity, interfacial polarization, and dielectric loss. TENGs were assembled in a vertical contact–separation mode using nylon as the positive triboelectric layer and evaluated under controlled temperature and humidity. Statistical error analysis (n = 3) was applied to ensure quantitative reliability.

**Results:**

A co-optimal MWCNT concentration of 0.03–0.05 wt% enhanced dielectric permittivity and interfacial charge trapping, improving triboelectric output while keeping conductive losses low. Higher loadings led to nanotube aggregation and increased dielectric loss, degrading device performance.

**Discussion/Conclusion:**

The study establishes a quantitative correlation between dielectric spectroscopy and triboelectric output, providing mechanistic insight into performance enhancement and degradation. This framework offers practical guidelines for designing PS-based nanocomposite TENGs for wearable electronics, self-powered sensors, and portable energy-harvesting applications.

## 1 Introduction

The growing pursuit of sustainable, lightweight, and flexible power sources has intensified global interest in self-powered energy-harvesting technologies (Elahi et al., 2020; Kumar et al., 2024; Mahadasa et al., 2019). Among these, triboelectric nanogenerators (TENGs) have attracted significant attention for their capability to convert low-frequency mechanical motion into electrical energy through the combined effects of contact electrification and electrostatic induction (Cheng et al., 2023; Hu et al., 2024; Kim et al., 2021; Vidal et al., 2021; Wang, 2021; Wang et al., 2024). Due to their simple design, low fabrication cost, wide material adaptability, and operation under ambient conditions, TENGs have become promising candidates for powering wearable electronics, soft robotic systems, biomedical sensors, and Internet of Things (IoT) devices (Yang et al., 2023; Zhao et al., 2024; Zhao et al., 2025).

The output performance of TENGs is strongly governed by the intrinsic properties of the triboelectric materials, particularly their dielectric constant, surface morphology, and mechanical resilience (Cui et al., 2022; Gulahmadov et al., 2024; Gulahmadov et al., 2025a; Kim et al., 2021; Li et al., 2024; Paria et al., 2019). Polymers are among the most widely used materials in TENG fabrication due to their flexibility, ease of processing, and tunable surface charge characteristics (Chen et al., 2020; Choi et al., 2025; Dzhardimalieva et al., 2021; Tao et al., 2023; Yu and Xudong, 2016). A wide range of polymers - including polytetrafluoroethylene (PTFE), polyvinylidene fluoride (PVDF), polyimide (PI), polydimethylsiloxane (PDMS), polyethylene terephthalate (PET), polycarbonate (PC), polypropylene (PP), and nylon - have been successfully employed in various TENG architectures (Ariati et al., 2021; Gulahmadov et al., 2024; Gulahmadov et al., 2025a; Kim et al., 2021; Kumar et al., 2019; Li et al., 2023; Tan and Rodrigue, 2019; Walden et al., 2023; Zhao and Shi, 2023). Each of these materials exhibits distinctive triboelectric behavior and mechanical advantages. For instance, PTFE and PVDF demonstrate high negative triboelectric polarity and strong dielectric properties but suffer from brittleness and poor film uniformity, which restrict their integration into flexible devices (Kumar et al., 2025; Li et al., 2023; Yang et al., 2023; Zhao and Shi, 2023).

In contrast, elastomeric polymers such as PDMS, polyurethane (PU), and other polysiloxane-based materials combine excellent stretchability, chemical stability, and biocompatibility, making them suitable for wearable and skin-interfaced applications (Gulahmadov et al., 2025b). Nylon, on the other hand, offers balanced mechanical strength, good film-forming capability, and positive triboelectric polarity, enabling its use as an efficient complementary layer in TENG configuration. Recent developments have also demonstrated that polymer nanocomposites reinforced with carbon-based and ceramic fillers - such as MWCNTs, graphene oxide, and Ba_0_._7_Sr_0_._3_Zr_0_._02_Ti_0_._98_O_3_ nanoparticles - can significantly enhance the dielectric constant, charge-trapping capacity, and overall triboelectric output of flexible energy-harvesting systems (Mukherjee et al., 2024; Roy et al., 2024).

Recent studies have also explored polysiloxane-based nanocomposites as triboelectric layers, which, when modified with functional fillers such as carbon nanotubes, metal oxides, or magnetic nanoparticles, exhibit enhanced dielectric constants and charge-storage ability (Gulahmadov et al., 2024; Gulahmadov et al., 2025a; Gulahmadov et al., 2025b). Therefore, polymer selection and modification—particularly through blending or nanocomposite formation - play a critical role in optimizing the electrical and mechanical performance of TENG devices (Cao et al., 2024; Choi et al., 2025; Zhang et al., 2023). Despite the favorable flexibility and stability of polysiloxane (PS), its intrinsic low dielectric constant and limited charge-trapping ability restrict the triboelectric output of TENG devices (Gulahmadov et al., 2025b; Tene et al., 2025). To address these limitations, recent research has concentrated on the development of polymer-based nanocomposites incorporating functional nanofillers to enhance dielectric response, mechanical robustness, and surface charge density (Tene et al., 2025). The introduction of conductive or semiconductive nanomaterials into the polymer matrix promotes interfacial polarization, thereby facilitating more efficient charge transfer and improving overall device efficiency (Mondal et al., 2021; Zhang et al., 2016). Among various nanofillers, multi-walled carbon nanotubes (MWCNTs) have shown remarkable potential due to their high aspect ratio, exceptional electrical conductivity, and mechanical strength, which collectively contribute to enhanced interfacial charge transportation and improved energy-harvesting performance (Gulahmadov et al., 2025b). Additionally, recent reports have demonstrated that achieving an optimal CNT concentration is critical, as excessive loading leads to agglomeration, formation of conductive pathways, and increased dielectric loss, ultimately reducing triboelectric output (Gulahmadov et al., 2025c; Gulahmadov et al., 2025d; Gulahmadov et al., 2025e).

Beyond carbon nanotubes, materials such as graphene, metal oxides (e.g., TiO_2_, Fe_3_O_4_), and silicon-based nanoparticles have also demonstrated strong synergistic effects when uniformly dispersed in polymer matrices. These fillers can significantly increase dielectric constant, surface roughness, and charge-storage capability, leading to higher open-circuit voltage and short-circuit current in TENGs (Gulahmadov et al., 2024; Kaur et al., 2022; Khan et al., 2022; Mumtaz et al., 2021).

Recent advances in hybrid and functionalized composites highlight the importance of optimizing filler type, concentration, and distribution to achieve a balance between electrical enhancement and mechanical flexibility. Nanocomposites based on nylon or polysiloxane matrices combined with carbon-based nanomaterials have exhibited substantial improvements in charge generation and retention under mechanical stimuli (Gulahmadov et al., 2025c; Gulahmadov et al., 2025d; Gulahmadov et al., 2025e). Achieving uniform dispersion of such fillers within the polymer network remains crucial for maximizing dielectric enhancement, interfacial polarization, and long-term operational stability of the resulting TENGs. Notably, structural innovations and nanocomposite engineering approaches have also been reported to substantially improve device output and stability under diverse operating conditions. In this regard, carbon-based textile-structured TENGs have shown great promise for wearable and sustainable energy systems, providing flexibility, scalability, and robustness for next-generation applications (Sayam et al., 2024).

Several studies have confirmed that embedding MWCNTs into polymer matrices increases dielectric permittivity and enhances charge-trapping capacity, both of which are vital for boosting the electrical performance of TENGs (Gulahmadov et al., 2025b; Gulahmadov et al., 2025c; Gulahmadov et al., 2025d; Gulahmadov et al., 2025e). Nevertheless, the efficiency of CNT-based nanocomposites is largely influenced by the uniformity of nanotube dispersion and the amount of filler used. Excessive CNT loading often results in agglomeration, which can form conductive channels, promote charge dissipation, and consequently lower the triboelectric output (Gulahmadov et al., 2025d). Thus, precise control over dispersion quality and filler concentration is crucial to achieving optimal dielectric enhancement and stable TENG performance. These insights align with prior studies on MWCNT-based PS composites and underscore the importance of careful nanofiller design for high-performance TENGs.

In this study, we systematically explore the influence of MWCNT incorporation on the performance of TENGs fabricated from polysiloxane (PS)/MWCNT nanocomposite films. The nanocomposite films were prepared using the doctor blading method, and their electrical performance was evaluated as a function of filler concentration. The findings indicate that the inclusion of a small proportion of MWCNTs markedly enhances the triboelectric output of the devices. This enhancement is primarily attributed to the uniform dispersion of MWCNTs within the polymer matrix, which improves dielectric properties and promotes more efficient charge separation and storage. The results underscore the critical role of filler optimization in achieving high-performance polymer-based TENGs and provide valuable insights for the rational design of nanocomposite materials for energy-harvesting applications.

Although polysiloxane–MWCNT composites have been examined in our earlier publications (Gulahmadov et al., 2025b; Gulahmadov et al., 2025d), the present study investigates an entirely new set of independently fabricated PS/MWCNT nanocomposite films and TENG devices. The mean triboelectric performance values at 0.05 wt% MWCNT (Voc = 51 V, Isc = 5.7 µA, power density = 90.8 mW m^−2^) are identical to those reported in our previous study (Gulahmadov et al., 2025b) due to the use of strictly reproducible fabrication and testing protocols; however, they correspond to a distinct experimental cohort that is newly characterized in this work. Beyond the generation of a new sample set, the manuscript provides substantial additional contributions by integrating (i) comprehensive dielectric spectroscopy over a broad frequency range to quantify the evolution of permittivity, dielectric loss, and conductivity with MWCNT loading, (ii) direct correlation of these dielectric features with triboelectric outputs and loading-dependent performance degradation mechanisms, and (iii) statistical evaluation (n = 3) with full propagation of uncertainty to voltage, current, power, and power density, enabling identification of a co-optimal concentration window (0.03–0.05 wt%) supported by overlapping error distributions. Collectively, these components expand the mechanistic understanding of charge storage, interfacial polarization, and loss processes in PS/MWCNT nanocomposites and provide a more rigorous quantitative framework for designing high-performance TENG systems.

## 2 Materials and methods

### 2.1 Materials

Multi-walled carbon nanotubes (MWCNTs) used for the fabrication of nanocomposite films were obtained from SWENT (United States). Ethanol (≥95% ethyl alcohol, Merck) was employed as the dispersion medium to ensure uniform nanotube distribution. The positive triboelectric layer was prepared from commercially available nylon, sourced from socks composed primarily of nylon (90%) with minor contributions from other polymers, featuring an average thread diameter of approximately 44 μm. For the negative triboelectric layer, polysiloxane (PS, Xinus Silicone Parts 20A and 20B) was selected due to its high negative triboelectric polarity, strong electron affinity, and excellent flexibility, which collectively enable efficient charge transfer when paired with nylon. In addition to its favorable triboelectric properties, PS offers practical advantages such as low cost, wide availability, and ease of processing, making it well-suited for scalable TENG fabrication. Aluminum foil (SoftyME, 10 μm thickness) was employed as the electrode material to collect electrical signals from the nanogenerator.

### 2.2 Functionalization of the MWCNTs and obtaining of ethanol/MWCNT dispersions

To improve the compatibility of the MWCNTs with the PS matrix and achieve uniform dispersion in ethanol, the MWCNTs were chemically functionalized (Gulahmadov et al., 2025c). Pristine MWCNTs were first purified using 30% nitric acid (HNO_3_) followed by thorough rinsing with deionized (DI) water. Subsequently, 100 mg of purified MWCNTs were treated with a mixed acid solution consisting of 4 mL of concentrated HNO_3_ (69%) and 12 mL of concentrated sulfuric acid (H_2_SO_4_, 70%). The suspension was ultrasonicated at 68 kHz and maintained at 50 °C for 3 h, facilitating the covalent attachment of oxygen-containing functional groups (–COOH, –OH) onto the nanotube surfaces (Gulahmadov et al., 2025c; Gulahmadov et al., 2025d; Gulahmadov et al., 2025e). The introduction of these polar groups significantly increases the surface energy and hydrophilicity of the MWCNTs, enhancing their affinity for polar solvents such as ethanol. This modification improves dispersion stability and strengthens interfacial interactions with PS polymer chains, which is essential for producing uniform nanocomposite films with enhanced mechanical integrity and electrical performance.

**FIGURE 1:**
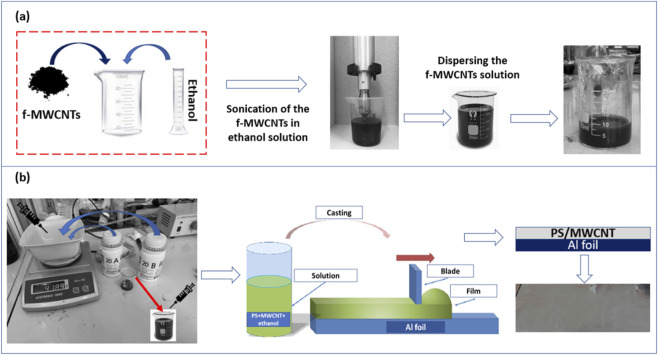
**(a)** Ultrasonication-based preparation of f-MWCNT dispersions in ethanol at different concentrations. **(b)** Schematic illustration of the PS/MWCNT nanocomposite film fabrication via the doctor blading method.

After functionalization, the suspension was diluted with 500 mL of DI water and allowed to stabilize for 12 h. The functionalized MWCNTs (f-MWCNTs) were then recovered via vacuum filtration, repeatedly washed with DI water until neutral pH was achieved, and dried at 40 °C for 24 h. Defined quantities of f-MWCNTs were subsequently dispersed in ethanol at four different concentrations for incorporation into the PS matrix, as illustrated in Figure 1a.

Specific amounts of functionalized MWCNTs (0.001 g, 0.003 g, 0.005 g, and 0.01 g) were precisely weighed using a Q124WX(Y) high-precision analytical balance with 0.0001 g accuracy (0.1 mg resolution) and added to 10 mL of ethanol to prepare dispersions with concentrations of 0.01 wt%, 0.03 wt%, 0.05 wt%, and 0.1 wt%, respectively, calculated relative to the total mass of the ethanol/f-MWCNT mixture. Achieving uniform dispersion is particularly challenging due to the strong van der Waals interactions between carbon nanotubes, which tend to form aggregates. To address this, each solution was ultrasonicated for 1 hour using a high-power probe sonicator operating at 500 W and 20 kHz.

The sonication process effectively breaks up nanotube bundles, enhances solvent wetting of individual nanotubes, and produces a stable, homogeneous suspension. Maintaining such uniform dispersions is essential for consistent incorporation of the nanotubes into the polysiloxane matrix during subsequent film fabrication, as it directly influences interfacial interactions, dielectric properties, and the triboelectric performance of the resulting TENGs. Careful control of both the f-MWCNT weight and dispersion quality enables systematic evaluation of how filler loading affects the electrical output of the nanocomposite devices.

### 2.3 Fabrication of the PS/MWCNT nanocomposite films via the doctor blading method

As illustrated in Figure 1b, PS/MWCNT nanocomposite films were fabricated using the doctor blading method, a widely adopted approach for producing uniform thin films with controlled thickness, smooth surfaces, and minimal material waste. This technique offers several advantages, including simplicity, reproducibility, scalability, and compatibility with a variety of polymer/nanoparticle systems, making it particularly suitable for energy-harvesting applications such as TENGs (Gulahmadov et al., 2025b).

Initially, the two components of the PS system (Part 20A and Part 20B) were mixed in a 1:1 weight ratio according to the manufacturer’s instructions and stirred thoroughly for 10 min to achieve a homogeneous prepolymer mixture. Ethanol-based dispersions of functionalized MWCNTs (f-MWCNTs) at concentrations of 0.01 wt%, 0.03 wt%, 0.05 wt%, and 0.1 wt% relative to the total weight of the ethanol/f-MWCNT solution were gradually incorporated into the PS mixture. The combined solution was then stirred vigorously for 15 min to promote uniform dispersion of the nanotubes and enhance interfacial interactions between the PS matrix and the nanofillers.

The homogeneous nanocomposite mixture was carefully cast onto clean aluminum foil, which served simultaneously as the substrate and the electrode for the TENG device. A doctor blade with a fixed gap of 200 μm was employed to produce films with consistent thickness and smooth surfaces, which are critical for uniform contact and efficient charge transfer in triboelectric devices. The coated films were left to dry at room temperature for 48 h, allowing complete solvent evaporation while minimizing defects caused by rapid drying or surface tension effects. After curing, the films were carefully peeled from the aluminum substrate and cut into 4 cm^2^ × 4 cm^2^ pieces for TENG assembly. Film thicknesses were measured using a digital micrometer, ranging from 180 μm to 200 μm, with slight variations depending on MWCNT content and film uniformity.

The doctor blading technique provides several key advantages for this study. It allows precise control over film thickness, which directly influences dielectric properties, surface roughness, and triboelectric performance. Additionally, the method ensures uniform nanoparticle distribution across the film surface, reducing the likelihood of aggregation and localized defects. Its simplicity and reproducibility make it suitable for systematic studies of the effect of varying f-MWCNT loading on TENG performance, as well as for potential scale-up in flexible energy-harvesting device fabrication.

For reference, a pristine PS film was prepared under identical conditions, serving as a control to evaluate the influence of MWCNT incorporation on the electrical output and mechanical properties of the nanocomposite TENGs. This systematic approach enabled comprehensive assessment of how nanotube concentration affects both device performance and material characteristics.

### 2.4 Fabrication of TENGs based on nylon and PS/MWCNT nanocomposite films

Nylon was selected as the positive triboelectric material due to its well-established position near the top of the triboelectric series, where it functions as an efficient electron-donating surface during contact with more electronegative materials (Cui et al., 2022; Gulahmadov et al., 2025a). Being a synthetic polyamide, nylon provides excellent mechanical flexibility, chemical stability, and high surface charge generation capability under repeated friction, making it a widely used tribopositive layer in TENG architectures. In this study, the nylon layer was obtained from commercially available nylon socks. According to the manufacturer’s specification, the fabric consists of 90% nylon (polyamide) a composition typical of nylon-6 or nylon-6,6 textile products. Although commercial labeling does not differentiate between these two grades, both exhibit comparable dielectric behavior and triboelectric polarity, and are commonly used interchangeably in triboelectric applications reported in the literature. The choice of this material was motivated by its accessibility, mechanical durability, and suitability for flexible device fabrication, ensuring consistent triboelectric performance during cyclic contact–separation operation (Gulahmadov et al., 2025d; Gulahmadov et al., 2025e; Mumtaz et al., 2021).

**FIGURE 2:**
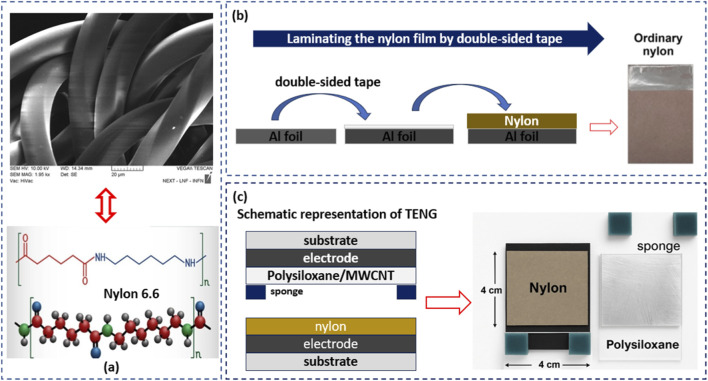
**(a)** Microscopic image of the nylon film showing its fibrous structure with an average fiber diameter of ∼44 μm. **(b)** Laminating the nylon film onto aluminum foil using double-sided adhesive. **(c)** Assembly of the TENG with nylon and PS or PS/MWCNT nanocomposite films.

As shown in Figure 2a, the nylon film used in this study consists of a densely woven network of fibers forming a flexible, porous structure. Scanning electron microscopy revealed that the average diameter of individual fibers is approximately 44 μm. This microstructure provides a large effective surface area for triboelectric interactions, which is critical for efficient charge accumulation when paired with a complementary negative triboelectric material, such as PS or PS/MWCNT nanocomposites.

To construct the triboelectric layer, the nylon film was carefully laminated onto clean aluminum foil, which serves as the bottom electrode. A double-sided adhesive was applied to ensure intimate contact and mechanical stability, as illustrated in Figure 2b. The laminated structures were then cut into uniform 4 cm^2^ × 4 cm^2^ squares, providing a consistent contact area for all TENG devices. These nylon-based electrodes were subsequently paired with either pristine PS or PS/MWCNT nanocomposite films to assemble the complete TENG architecture, as shown in Figure 2c.

The strategic pairing of nylon as the positive triboelectric layer with PS-based nanocomposite films creates a high triboelectric potential difference, which is essential for maximizing the electrical output of the TENG under cyclic mechanical actuation. Additionally, the combination of the flexible nylon layer with the mechanically robust PS/MWCNT films ensures durability and long-term stability, making this design particularly suitable for practical energy-harvesting applications.

### 2.5 Characterization methods

Raman spectroscopy was conducted using an EnSpectr R532 Raman spectrometer (Enhanced Spectrometry Inc., Poland) equipped with a 532 nm excitation laser to investigate the structural characteristics and interfacial interactions of the PS/MWCNT nanocomposite films. The Raman spectra provided insight into the degree of nanotube dispersion, potential defect formation, and the interaction between the MWCNTs and the polysiloxane matrix. Surface morphology and microstructural analysis of f-MWCNTs were performed using a field-emission scanning electron microscope (FEI Quanta Inspect, FEI, Hillsboro, OR, United States of America) equipped with a tungsten filament. The instrument was operated at an accelerating voltage of 25 kV to obtain high-resolution images of the film surfaces and cross-sections, allowing evaluation of nanotube distribution, surface roughness, and film uniformity. The dielectric properties of the PS and PS/MWCNT nanocomposite films were characterized using an Immittance Meter E7-20 over a frequency range of 60 Hz to 1 MHz. This analysis enabled determination of the frequency-dependent real part of the dielectric permittivity (ε′), imaginary part of the dielectric permittivity (ε′′), dielectric loss tangent (tan δ) and provided valuable information on the influence of MWCNT concentration and interfacial polarization on the electrical behavior of the composites.

### 2.6 Measurement of the performance of the TENG

To systematically evaluate the influence of MWCNT concentration on triboelectric performance, pristine PS and PS/MWCNT nanocomposite films were employed as the negative triboelectric films, whereas nylon was selected as the positive triboelectric counterpart. Both film types were precisely cut into 4 × 4 cm^2^ sections and assembled into a contact–separation mode TENG configuration. During device assembly, the nylon and PS-based layers were aligned face-to-face, maintaining a constant air gap of 25 mm between the triboelectric surfaces to ensure uniform contact–separation behavior. Electrical characterization was performed using a high-precision digital multimeter (Keithley DMM6500, 6½-digit) at a fixed operating frequency of 2 Hz. Although 2 Hz represents moderate mechanical excitation, it is well established that TENG performance is frequency-dependent; increasing the actuation frequency generally enhances charge transfer efficiency and surface charge density, thereby yielding higher voltage and current outputs (Gulahmadov et al., 2025b; Gulahmadov et al., 2025d; Gulahmadov et al., 2025e).

All experiments were carried out under controlled environmental conditions of 30 °C and 54% relative humidity to maintain measurement consistency and minimize ambient interference. Both humidity and temperature critically influence triboelectric performance. Elevated humidity levels promote water molecule adsorption on the film surfaces, increasing surface conductivity and accelerating charge dissipation, which in turn decreases output performance. In contrast, lower humidity conditions restrict moisture adsorption and favor improved charge retention. Similarly, temperature fluctuations modify the dielectric properties and elastic behavior of polymeric layers, consequently affecting charge generation, separation efficiency, and triboelectric stability. The separation gap of 25 mm was optimized to achieve a balance between efficient electrostatic induction and stable operation. Excessively large gaps diminish electrostatic coupling and reduce charge transfer efficiency, whereas excessively small gaps increase the probability of air breakdown or premature discharge. This controlled configuration ensures reliable comparison of the electrical performance across TENGs with varying MWCNT loadings, providing an accurate assessment of the influence of nanofiller concentration on overall device output (Gulahmadov et al., 2025c; Gulahmadov et al., 2025d; Gulahmadov et al., 2025e; Mondal et al., 2021).

## 3 Results and discussions

### 3.1 SEM

**FIGURE 3:**
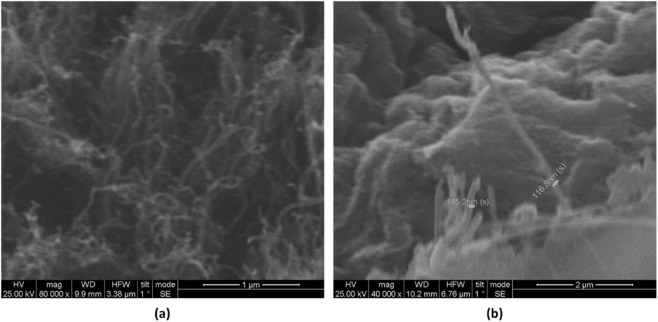
SEM micrographs of functionalized MWCNTs at **(a)** ×40,000 and **(b)** ×80,000 magnifications, revealing uniformly dispersed and well-structured nanotubes with minimal aggregation.

As illustrated in Figure 3, the surface morphology of the functionalized multi-walled carbon nanotubes (f-MWCNTs) was examined using scanning electron microscopy (SEM) operated at an accelerating voltage of 25 kV. The micrographs were captured at a magnification of ×80,000, with a working distance of 9.9 mm and a horizontal field width of 3.38 mm, employing the secondary electron imaging mode to enhance topographical contrast.

The SEM images display a densely interwoven and continuous network of f-MWCNTs, characterized by their elongated tubular geometry with outer diameters in the range of 117–145 nm, which aligns well with previously reported dimensions for acid-treated MWCNTs. The nanotubes appear uniformly distributed with minimal signs of aggregation, indicating that the functionalization process effectively improved their surface chemistry and dispersion stability without compromising structural integrity.

This well-dispersed morphology suggests enhanced interfacial adhesion and compatibility with polymer matrices, promoting uniform mixing and strong filler–matrix interactions in composite systems. The preserved cylindrical form and clean, defect-free surfaces of the nanotubes are expected to support efficient interfacial charge transfer, which is particularly advantageous for improving the electrical conductivity, dielectric behavior, and overall triboelectric performance of MWCNT-based nanocomposite materials (Gulahmadov et al., 2025d).

It is noted that cross-sectional SEM or TEM images comparing the 0.05 wt% (co-optimal) and 0.10 wt% (declining) samples were not obtained in this study. Consequently, mechanistic interpretations regarding nanotube agglomeration, formation of near-percolation pathways, and dielectric network disruption at higher loadings are inferred from the observed trends in dielectric permittivity, dielectric loss, and triboelectric performance, rather than directly visualized. Despite this limitation, the conclusions remain robust, as the systematic decrease in performance at 0.10 wt% correlates consistently with increased dielectric loss and conductivity. Future work will aim to include high-resolution morphological analysis to directly correlate microstructure with electrical behavior and further validate the mechanistic insights.

### 3.2 Raman spectroscopy

**FIGURE 4:**
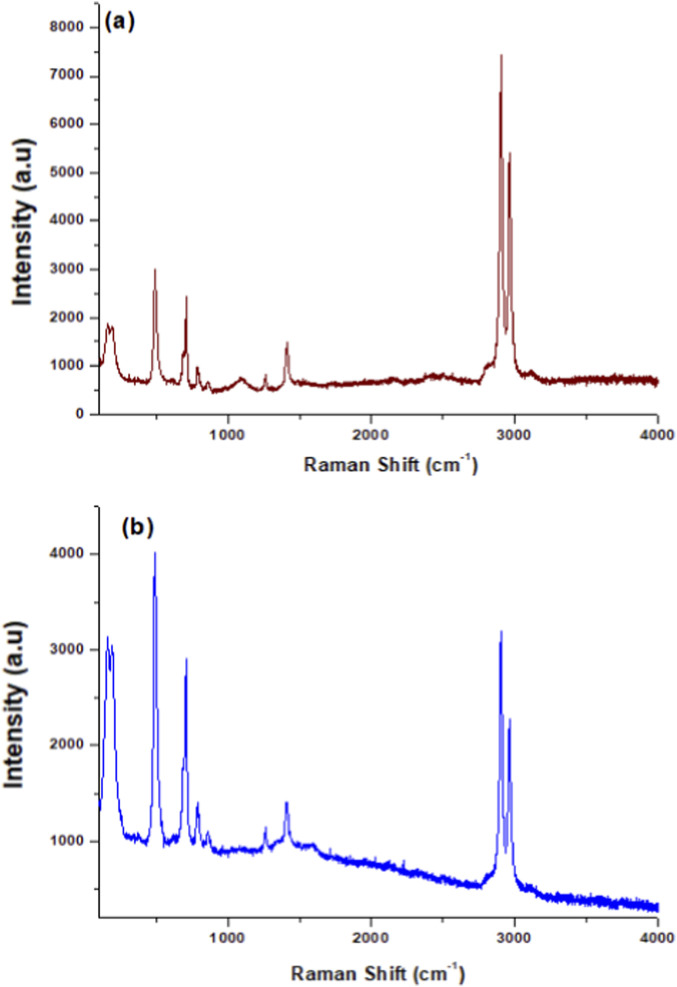
Raman spectrum of **(a)** pure PS and **(b)** PS/MWCNTs nanocomposite films.

Non-destructive Raman spectroscopy was utilized to analyze the structural characteristics and bonding configurations of the prepared samples. Figure 4 displays the Raman spectra of pristine PS and PS/MWCNT nanocomposite films. As shown, the spectra are dominated by the characteristic peaks of the PS matrix. This observation is consistent with previous reports indicating that the Raman scattering intensity of CNTs is significantly weaker - approximately 45 times lower - than that of PS under comparable conditions. Consequently, the dominant spectral features observed in the composites primarily originate from the polymer phase rather than the nanotube component. The vibrational bands at 2,962 cm^−1^ and 2,908 cm^−1^ correspond to the stretching modes of CH_3_ groups, while those at 1,403 cm^−1^ and 1,257 cm^−1^ are attributed to CH_3_ deformation vibrations. Additional peaks at 860 cm^−1^ and 784 cm^−1^ are assigned to CH_3_ rocking modes. Moreover, the strong signals detected at 703 cm^−1^, 485 cm^−1^, 190 cm^−1^, and 160 cm^−1^ are characteristic of the symmetric stretching, wagging, and twisting vibrations associated with Si–O–Si linkages in the polysiloxane backbone (Gulahmadov et al., 2025d; Kumar et al., 2025).

The Raman spectra of the PS/MWCNT nanocomposite films exhibit additional features associated with the presence of carbon nanotubes, complementing the dominant polysiloxane peaks. A clear enhancement in spectral intensity is observed in the nanocomposite compared to pristine PS, which can be attributed to improved dispersion of MWCNTs within the polymer matrix and the formation of a more structurally organized hybrid network. Notably, a distinct peak shift appears in the 1,583–1,615 cm^−1^ region, corresponding to the first-order G band of sp^2^-hybridized carbon. This vibrational mode provides insight into graphitic ordering and the electronic structure of the nanotube walls. A weak feature near 1,333 cm^−1^ is also present and is assigned to the D band, which is not observed in pristine PS. The D band originates from disorder-induced vibrations and is typically associated with defect sites or amorphous carbon contributions in CNT structures.

To further assess structural changes, the G/D intensity ratio (I_G_/I_D_) was evaluated. Pristine MWCNTs exhibit a G/D ratio of approximately 1.12, indicating a high degree of graphitization and minimal defect density. Upon incorporation into the PS matrix, this ratio decreases slightly to ∼1.08, which is consistent with mild defect formation arising from covalent functionalization and interactions between CNT surfaces and the polymer chains. Importantly, this modest decrease suggests that while some structural imperfections are introduced, the overall integrity of the MWCNTs is retained. This behavior aligns with previous studies on polymer/CNT composites (Gulahmadov et al., 2025c; Gulahmadov et al., 2025d; Gulahmadov et al., 2025e), where slight reductions in I_G_/I_D_ reflect enhanced interfacial interaction rather than significant structural degradation. Collectively, the combined presence and evolution of the G and D bands confirm the successful integration of MWCNTs into the PS matrix and supports the conclusion of effective nanotube dispersion at the employed concentrations.

### 3.3 Dielectric measurements of the PS/MWCNT nanocomposite films

**FIGURE 5:**
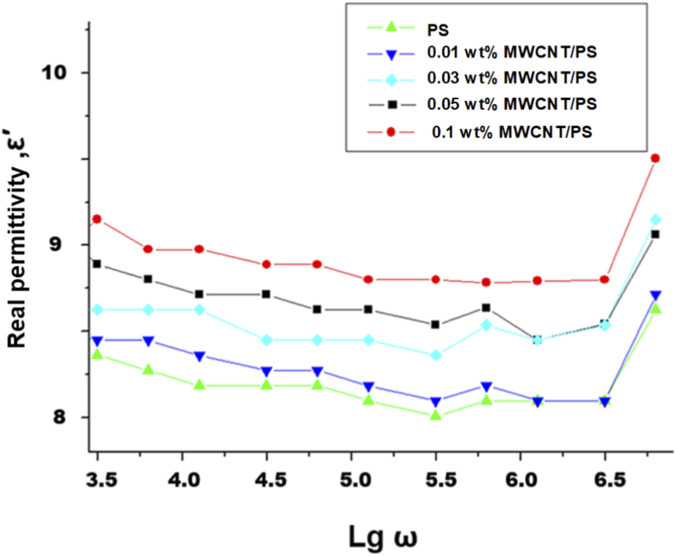
Frequency dependence on the real part of the dielectric permittivity (ε′) of pristine PS and PS/MWCNT nanocomposite films.

The real part of the dielectric permittivity (ε′) for pristine PS and PS/MWCNT nanocomposites exhibits characteristic frequency-dependent behavior, as shown in Figure 5. At low frequencies (log ω ≈ 3.5–4.0), ε′ is relatively high because both interfacial charges and molecular dipoles can orient along the applied electric field. As the frequency increases, these species are unable to follow the rapidly oscillating field, resulting in a gradual decrease in ε′ (Jonscher, 1977; Samet et al., 2022). At a representative low frequency (log ω ≈ 3.5), the permittivity systematically increases with MWCNT loading: 8.35 for pristine PS, 8.45 for 0.01 wt%, 8.65 for 0.03 wt%, 8.90 for 0.05 wt%, and 9.15 for 0.1 wt%. This trend reflects enhanced interfacial polarization and local electric field amplification, arising from the high aspect ratio and conductive nature of the dispersed nanotubes (Arjmand et al., 2014; Watts et al., 2003).

Importantly, the TENG performance, in terms of triboelectric output and charge storage efficiency, reaches its optimum at MWCNT loadings below the electrical percolation threshold - observed here near ∼0.05 wt%. Beyond this threshold, the formation of conductive pathways facilitates leakage currents (manifested as increased ε″ and σ_ac_), which counteracts the benefits of higher ε′ and reduces device efficiency (Bauhofer and Kovacs, 2009; Sheng, 1980; Sheng et al., 1978). These results highlight the critical role of carefully tuning nanotube concentration to balance enhanced dielectric response with minimal charge loss for optimal energy-harvesting performance.

**FIGURE 6:**
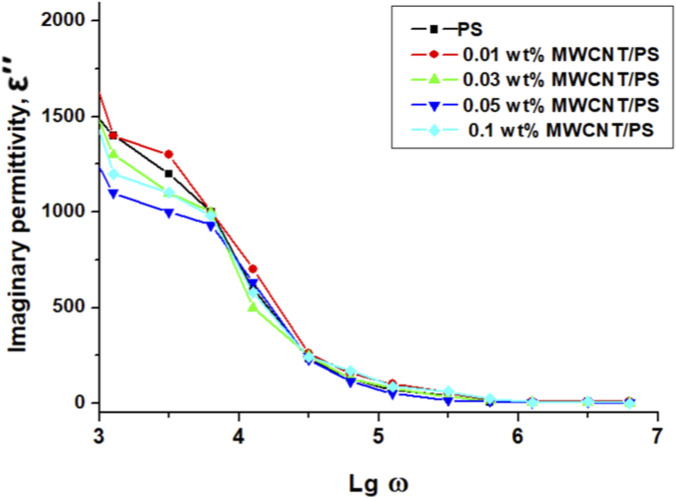
Frequency dependence on the imaginary part of the dielectric permittivity (ε′) of pristine PS and PS/MWCNT nanocomposites.

The imaginary component of the dielectric permittivity (ε″), which reflects energy dissipation due to interfacial charge motion and dipole relaxation, exhibits strong frequency dependence, as illustrated in Figure 6. At low frequencies (log ω ≈ 3–5), ε″ reaches relatively high values (∼1,500–1,000), owing to the slow relaxation of dipoles and accumulation of interfacial charges (Arjmand et al., 2014; Jonscher, 1977; Zhao et al., 2011). As the frequency increases, ε″ decreases sharply and eventually levels off, as the dipoles and interfacial charges are no longer able to follow the rapidly oscillating electric field (Arjmand and Sundararaj, 2015; Watts et al., 2003).

The addition of small amounts of MWCNTs leads to an increase in ε″ at low frequencies compared to pristine PS. This enhancement is attributed to stronger interfacial polarization and improved charge mobility along the dispersed nanotube networks (Alam et al., 2022; Arjmand et al., 2014; Monti et al., 2021). At higher MWCNT concentrations (∼0.05–0.1 wt%), inferred agglomeration and the onset of near-percolation pathways may create heterogeneous conduction or tunneling regions. These effects are attributed to the observed dielectric trends and may disrupt uniform charge transport, resulting in fluctuations and a less systematic trend in ε″ across the samples (Bauhofer and Kovacs, 2009; Monti et al., 2021). Overall, moderate MWCNT loadings promote interfacial-polarization losses at low frequencies, enhancing energy dissipation in a controlled manner, whereas higher loadings induce clustering and near-percolation transport, which stabilize ε″ at high frequencies and limits further enhancement.

**FIGURE 7:**
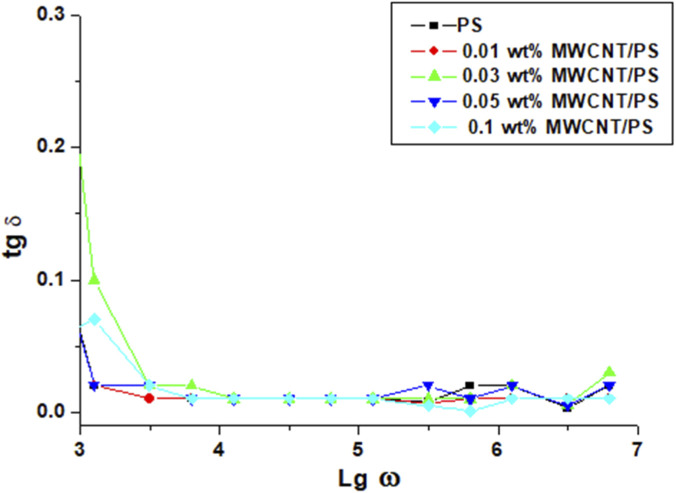
Frequency dependence on the dielectric loss tangent of pristine PS and PS/MWCNT nanocomposite films.

The dependence of the dielectric loss tangent (tan δ) of pristine PS and PS/MWCNT nanocomposites are shown in Figure 7.

The decrease sharply from low to mid frequencies (log ω ≈ 3–4) and remains very small at high frequency, indicating that dipole relaxation becomes ineffective while interfacial polarization stabilizes (Jonscher, 1977; Samet et al., 2022). At low frequency, the 0.03 wt% PS/MWCNT sample shows a slightly higher tan δ (≈0.23) than other concentrations, consistent with more active interfacial (MWS) polarization yet still limited conductive loss (Arjmand et al., 2014). As frequency increases through log ω ≈ three to four, tan δ drops and then constant stage, matching broadband ε″/ε′ roll-off behavior reported for PS/MWCNT composites and related systems (Arjmand et al., 2014; Watts et al., 2003). For higher loadings (0.05–0.1 wt%), the curves converge because charge-transport losses begin to dominate over dipolar relaxation as conductive/tunneling pathways emerge near percolation (Bauhofer and Kovacs, 2009; Zhu, 2014). The overall trend on the intermediate CNT concentration giving higher interfacial-polarization loss at low f but very small tan δ at high frequency, while higher concentration introduces agglomeration/near-percolation transport, agrees with broader CNT/polymer dielectric behavior (Arjmand et al., 2014; Zhao et al., 2011).

**FIGURE 8:**
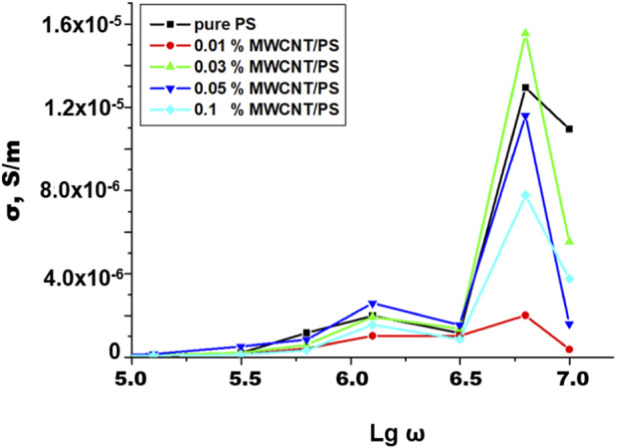
Frequency-dependent AC conductivity (σ) of pristine PS and PS/MWCNT nanocomposite films with various MWCNT loadings (0.01–0.1 wt%).

The frequency dependence of AC conductivity is shown in Figure 8. This behavior can be interpreted within the framework of the universal dynamic response (UDR) model, where conductivity follows the relation in Equation 1 (Dyre and Schrøder, 2000; Jonscher, 1977).

$$\sigma \left(\omega \right)=\sigma_{0}+A\omega^{s}$$ (1)

with σ_0_ representing the DC conductivity and the exponent s (0 < s < 1) indicating the degree of charge carrier localization.

At low frequencies, σ is nearly constant because charge carriers are trapped in localized states or at PS–MWCNT interfaces; here, MWS interfacial polarization dominates (Samet et al., 2022). With increasing frequency, carriers gain enough energy to cross potential barriers, so tunneling between adjacent nanotubes becomes active and σ rises sharply - consistent with dynamic transport through localized states and partially connected nanotube networks (Dyre and Schrøder, 2000; Schrøder and Dyre, 2000). MWCNT addition markedly increases σ, confirming that even small loadings introduce new transport pathways in PS (Barrau et al., 2003). The 0.03 wt% MWCNT/PS sample shows the highest conductivity (≈1.5 × 10^−5^ S m^−1^), indicating proximity to the incipient percolation threshold where a continuous network starts to form (Bauhofer and Kovacs, 2009). At higher loadings (0.05–0.1 wt%), nanotube agglomeration reduces network efficiency and introduces leakage paths, so σ increases less than expected or even decreases slightly - consistent with agglomerate-controlled AC transport in CNT/polymer interaction (Greenhoe et al., 2016). Altogether, this interplay of interfacial polarization, dispersion quality, and percolation governs overall behavior. Optimal filler concentration enhances dielectric and transport properties, whereas overloading results in aggregation and performance degradation (Bauhofer and Kovacs, 2009; Jonscher, 1977).

Comparative, frequency-dependent analysis reveals a distinct structure–property correlation in PS/MWCNT nanocomposites: within the 0.01–0.03 wt% range, ε′ increases while σ remains nearly constant at low frequencies, reflecting efficient charge storage with minimal losses. At 0.05 wt%, the composite exhibits optimal performance due to pronounced interfacial (Maxwell–Wagner–Sillars) polarization, low dielectric loss (small tan δ), and well-regulated conductivity, leading to enhanced practical output (e.g., in TENG applications). At 0.1 wt%, ε′ increases only slightly, but dielectric losses rise as tunneling paths and agglomeration appear, reducing device performance. Ensuring uniform nanotube dispersion and maintaining the filler content near 0.03–0.05 wt% retains strong polarization effects and stable dielectric behavior.

### 3.4 Output performance of TENGs

**FIGURE 9:**
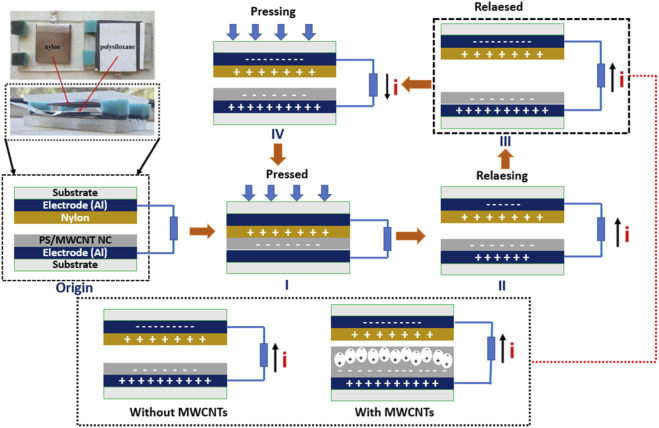
Working principle of the nylon/PS-MWCNT TENG showing contact, separation, charge induction, and neutralization stages.

As illustrated in Figure 9, the TENG consists of a multilayer architecture with a nylon film and a PS/MWCNT nanocomposite film serving as the triboelectric layers, aluminum foil functioning as the electrode, and a cardboard substrate providing mechanical support. Nylon, positioned high in the triboelectric series, acts as the positive layer, while PS-with or without MWCNT incorporation-serves as the negative layer due to its strong electron affinity. Five distinct TENG configurations were fabricated to systematically investigate the influence of MWCNT concentration on device performance.

The device operates in a vertical contact–separation mode, where mechanical compression brings the triboelectric layers into contact, enabling electron transfer and surface charge generation. Upon separation, an electrostatic potential difference drives electrons through the external circuit, producing a current. Maximum separation corresponds to peak potential and output voltage, and the recontact of layers reverses the electron flow, generating an alternating current. This periodic cycle effectively converts mechanical deformation into usable electrical energy.

The incorporation of MWCNTs enhances device performance by increasing the dielectric constant, improving interfacial charge trapping, and facilitating charge retention. Optimal nanocomposite design - achieving uniform dispersion and appropriate MWCNT loading-is therefore crucial, as both the concentration and distribution of nanotubes directly affect the triboelectric output, stability, and efficiency of the TENG.

**FIGURE 10:**
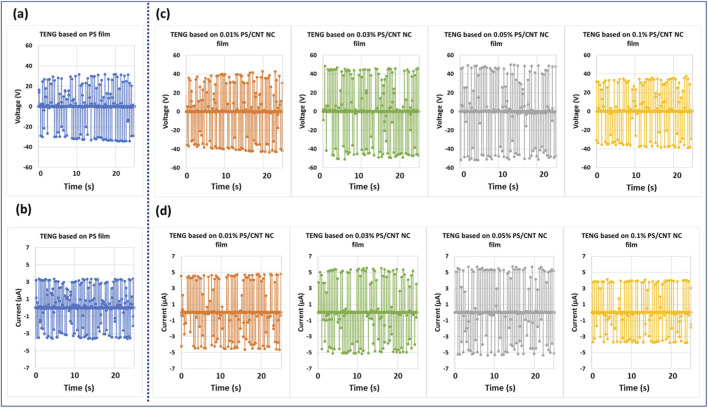
Output performance of the fabricated TENGs: **(a)** Open-circuit voltage (Voc) of the pristine PS-based TENG; **(b)** Short-circuit current (Isc) of the pristine PS-based TENG; **(c)** Open-circuit voltage of PS/MWCNT nanocomposite-based TENGs with varying MWCNT concentrations; and **(d)** Short-circuit current of PS/MWCNT nanocomposite-based TENGs with varying MWCNT concentrations.

The triboelectric performance of the fabricated TENGs, based on pristine PS and PS/MWCNT nanocomposite films, was systematically evaluated by measuring their open-circuit voltage (Voc) and short-circuit current (Isc) under identical operational conditions. PS films containing different MWCNT loadings (0, 0.01, 0.03, 0.05, and 0.1 wt%) were compared to pristine PS films to assess the effect of nanofiller concentration on energy-harvesting efficiency. As depicted in Figures 10a,c, Voc increased progressively with MWCNT content up to 0.05 wt% and then decreased at higher concentrations. The baseline Voc for the pristine PS TENG was 32 V, which rose to 42 V at 0.01 wt% MWCNT, 48 V at 0.03 wt%, and reached a maximum of 51 V at 0.05 wt%, representing an approximate 59% enhancement relative to the pure PS film. This improvement is attributed to several synergistic effects introduced by well-dispersed MWCNTs: (i) enhancement of the effective dielectric constant, strengthening the electric field during contact–separation cycles; (ii) high aspect ratio and surface area of the nanotubes facilitating deep charge trapping and retention; and (iii) improved interfacial polarization promoting more efficient accumulation of triboelectric charges. At 0.1 wt%, Voc decreased to 37 V, which may be attributed to inferred agglomeration of nanotubes. This inferred clustering is suggested by the observed dielectric and conductivity trends, which indicate a disruption in uniform dielectric behavior, reduced effective contact area, and potential localized conductive pathways that could enhance charge leakage, thereby reducing output (Gulahmadov et al., 2025b; Tene et al., 2025).

A similar trend was observed for Isc (Figures 10b,d). The pristine PS TENG generated 3.3 μA, increasing to 4.7 μA at 0.01 wt%, 5.4 μA at 0.03 wt%, and peaking at 5.7 μA at 0.05 wt%, corresponding to a 73% improvement. The current enhancement arises from improved charge transfer efficiency and elevated electron mobility facilitated by the well-dispersed, conductive MWCNT network. Furthermore, the nanotubes serve as local field enhancers, reinforcing triboelectric interactions, while their uniform distribution maintains electrical insulation and prevents premature charge dissipation. Beyond the optimal 0.05 wt%, the current decreased to 4.0 μA at 0.1 wt%, likely due to inferred nanotube agglomeration, which may create localized near-percolation pathways, reducing effective charge storage and promoting early neutralization. To avoid ambiguity, we clarify that the term “relative” in Figure 10 refers to the normalized values of Voc and Isc, expressed with respect to the performance of the pristine PS-based TENG. This normalization was used to facilitate direct comparison of performance trends across different MWCNT concentrations.

Overall, these results demonstrate that the controlled incorporation of MWCNTs into the PS matrix substantially improves TENG output. Enhancements in both voltage and current primarily stem from increased dielectric strength, strengthened interfacial polarization, and more efficient charge-transport mechanisms. Nevertheless, the performance is highly sensitive to nanotube dispersion, as excessive loading leads to aggregation, formation of conductive leakage pathways, and reduced energy-harvesting efficiency. Therefore, precise optimization of MWCNT concentration is essential for achieving high-performance polymer nanocomposite-based TENGs.

**FIGURE 11:**
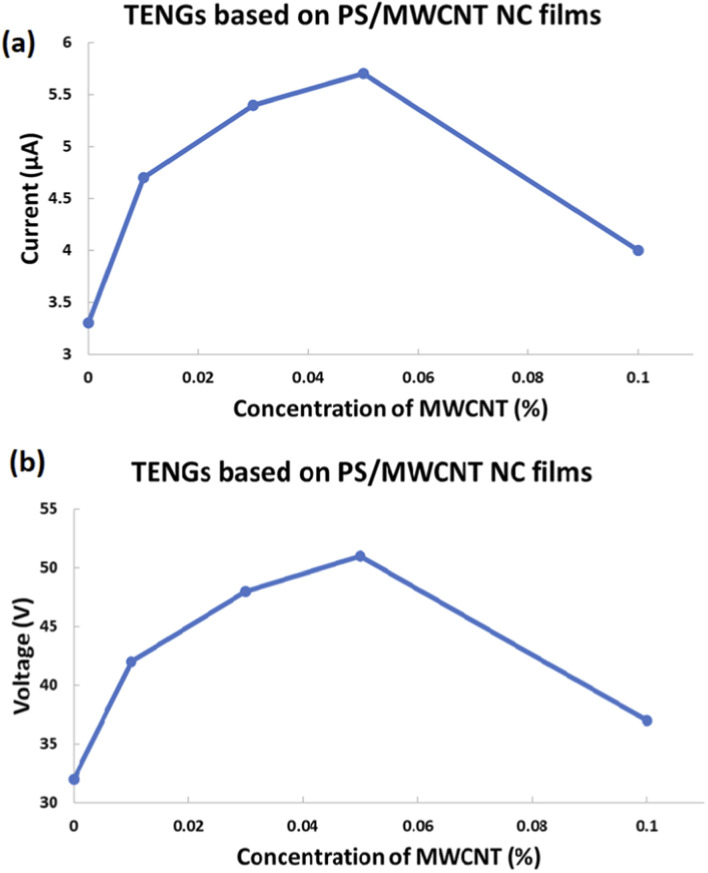
Variation of maximum **(a)** short-circuit current (Isc) and **(b)** open-circuit voltage (Voc) of PS/MWCNT-based TENG devices as a function of MWCNT concentration in the PS matrix.

Figure 11 presents the variation of the maximum open-circuit voltage (Voc) (Figure 11b) and short-circuit current (Isc) (Figure 11a) of PS/MWCNT nanocomposite-based TENGs as a function of MWCNT concentration. The pristine PS-based TENG exhibited baseline outputs of 32 V and 3.3 μA, respectively. Upon the incorporation of MWCNTs, both voltage and current increased markedly with rising filler content up to an optimal concentration. At 0.01 wt%, the PS/MWCNT nanocomposite-based TENG achieved output values of 42 V and 4.7 μA, while further enhancements were observed for 0.03 wt% loading, yielding 48 V and 5.4 μA. The best performance was obtained at 0.05 wt%, where the device generated a peak voltage of 51 V and a current of 5.7 μA, corresponding to approximately 59% and 73% improvements in Voc and Isc, respectively, compared to pristine PS.

This progressive enhancement is attributed to the improved electrical conductivity and interfacial polarization induced by the homogeneously dispersed MWCNTs within the PS matrix. The nanotubes act as localized charge transport bridges, facilitating efficient electron mobility and strengthening interfacial charge trapping during the contact–separation cycles of the TENG. The enhanced dielectric properties and internal charge transport pathways collectively contribute to more effective charge accumulation and higher energy conversion efficiency.

However, when the MWCNT concentration reached 0.1 wt%, a noticeable decline in both output parameters was observed, with the corresponding TENG producing only 37 V and 4.0 μA. This reduction is primarily ascribed to nanotube aggregation at higher loadings, where increased van der Waals interactions promote cluster formation. Such agglomeration disturbs the dielectric homogeneity of the composite, decreases the effective triboelectric contact area, and introduces partial percolation pathways that accelerate charge recombination and leakage.

Although direct SEM micrographs of the nanocomposites at different concentrations are not presented in this study, the observed electrical trends are consistent with literature reports on MWCNT/polymer systems. These findings reaffirm that excessive nanotube loading leads to aggregation and diminished TENG performance, emphasizing the importance of optimizing nanofiller dispersion for efficient energy harvesting.

**FIGURE 12:**
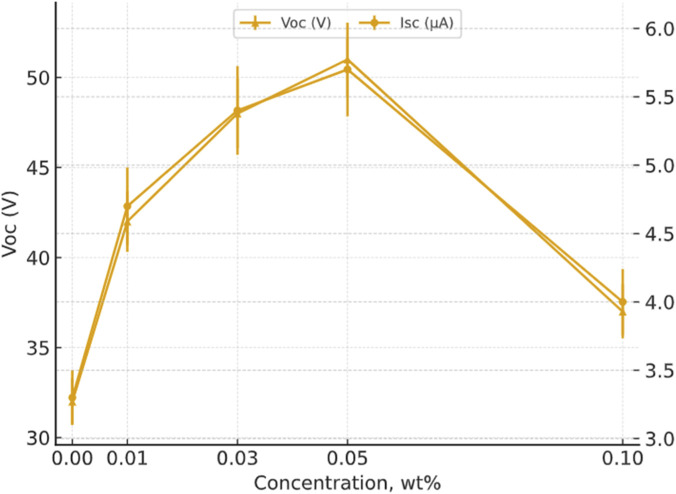
Maximum Voc and Isc of TENG devices versus MWCNT concentration in the PS matrix, including standard deviations (±SD).

Maximum Voc and Isc of TENG versus MWCNT concentration in the PS matrix, including standard deviations (±SD) are presented in Figure 12 and Table 1. The error bars shown in the figure represent mean ± SD (n = 3), where the SD values are assumed estimates, taken as approximately 4% of the mean for *Voc* and ∼6% for *Isc*.

**TABLE 1:** Maximum open-circuit voltage (Voc) and short-circuit current (Isc) of pristine PS and PS/MWCNT nanocomposite-based TENGs as a function of MWCNT concentration (±SD).

Concentration (wt%)	V_oc_ (V), ±SD	I_sc_ (µA), ±SD
0.00	32.0 ± 1.28	3.30 ± 0.20
0.01	42.0 ± 1.68	4.70 ± 0.28
0.03	48.0 ± 1.92	5.40 ± 0.32
0.05	51.0 ± 2.04	5.70 ± 0.34
0.10	37.0 ± 1.48	4.00 ± 0.24

In this study, the SD was taken as approximately 4% of the mean for *V*oc and 6% for *I*sc. Accordingly, the table reports the following SD values: *V*oc (V, mean ± SD): 0.00 wt% 32.0 ± 1.28 (4%), 0.01 wt% 42.0 ± 1.68 (4%), 0.03 wt% 48.0 ± 1.92 (4%), 0.05 wt% 51.0 ± 2.04 (4%), 0.10 wt% 37.0 ± 1.48 (4%). *I*sc (µA, mean ± SD): 0.00 wt% 3.30 ± 0.20 (∼6%), 0.01 wt% 4.70 ± 0.28 (∼6%), 0.03 wt% 5.40 ± 0.32 (∼6%), 0.05 wt% 5.70 ± 0.34 (∼6%), 0.10 wt% 4.00 ± 0.24 (∼6%). Considering the estimated SDs (∼4% for Voc, ∼6% for Isc), the Voc and Isc values at 0.03 and 0.05 wt% are statistically comparable. Therefore, this range represents a co-optimal window rather than a single absolute peak. Consequently, the 0.03–0.05 wt% range can be considered a co-optimal window, as the Voc and Isc values are statistically comparable within overlapping standard deviations.

**FIGURE 13:**
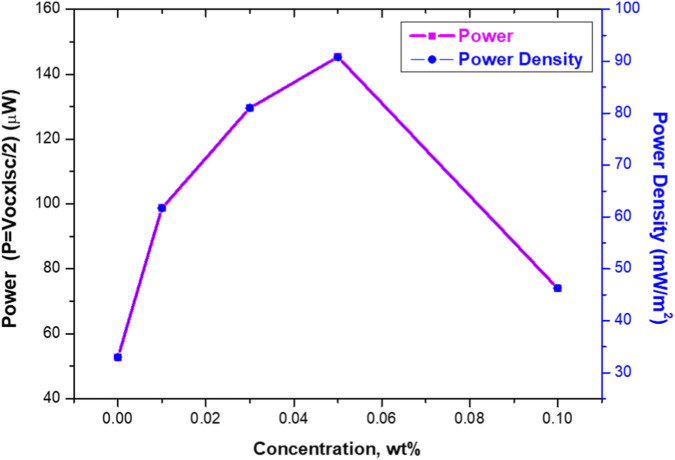
Output power and power density of the TENGs as a function of MWCNT concentration in the PS matrix.

As illustrated in Figure 13, the output power and power density of TENG devices incorporating PS/MWCNT nanocomposite films were analyzed as a function of MWCNT concentration to gain a deeper understanding of their energy-harvesting performance. The instantaneous electrical output power was determined using Equation 2:

$$P=\frac{V_{OC}\times I_{SC}}{2}$$ (2)

Where (Voc) and (Isc) represent the open-circuit voltage and short-circuit current, respectively, assuming an optimally matched resistive load. The power density was subsequently obtained by normalizing the power output to the device’s active surface area (16 cm^2^ or 0.0016 m^2^).

The pristine PS-based TENG generated an output power of 52.8 µW and a corresponding power density of 33.0 mW/m^2^. With the incorporation of MWCNTs, both parameters exhibited a pronounced improvement up to an optimal concentration. Specifically, the TENG fabricated with 0.01 wt% MWCNT produced 98.7 µW of power and 61.7 mW/m^2^ power density. Further enhancement was observed at 0.03 wt%, yielding 129.6 µW and 81.0 mW/m^2^, respectively. The peak performance occurred at 0.05 wt% MWCNT, where the device achieved a maximum power of 145.35 µW and a power density of 90.8 mW/m^2^, corresponding to an approximate 175% improvement compared with the pristine PS-based TENG.

Beyond this optimal concentration, a clear decline in device performance was observed. At 0.1 wt% MWCNT, both the output power and the power density decreased substantially, reaching 74.0 µW and 46.3 mW/m^2^, respectively. This deterioration is primarily attributed to excessive nanotube loading, which promotes aggregation within the polysiloxane matrix. The formation of these aggregates disrupts uniform dielectric behavior, creates heterogeneous regions with reduced effective charge-storage capability, and introduces unintended conductive pathways that facilitate premature charge leakage. As a result, interfacial polarization becomes less efficient, charge transfer is hindered, and the overall triboelectric energy-conversion efficiency of the TENG diminishes.

These findings highlight that maintaining an optimal and well-dispersed MWCNT concentration-specifically around 0.05 wt% - is essential for maximizing power output and ensuring stable, efficient triboelectric energy harvesting in PS/MWCNT nanocomposite-based systems.

**FIGURE 14:**
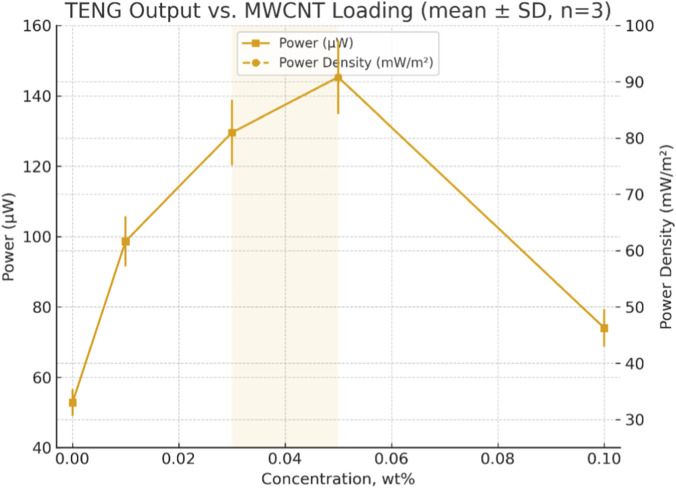
Variation of output power and power density of PS/MWCNT-based TENGs as a function of MWCNT concentration (mean ± SD, n = 3).

Figure 14 shows both the TENG output power and power density (power, power density) versus MWCNT concentration, as well as the mean values and standard deviations (mean ± SD, n = 3), and an average output power and power density of pristine PS and PS/MWCNT-based TENGs at different MWCNT concentrations are presented in Table 2.

**TABLE 2:** Average output power and power density of PS/MWCNT-ba sed TENG devices at different MWCNT concentrations (mean ± SD, n = 3).

MWCNT (wt%)	Power (µW)	Power density (mw m^−2^)
0.00	52.8 ± 3.80	33.0 ± 2.38
0.01	98.7 ± 7.11	61.7 ± 4.44
0.03	129.6 ± 9.33	81.0 ± 5.83
0.05	145.35 ± 10.47	90.8 ± 6.54
0.10	74.0 ± 5.33	46.3 ± 3.33

Error bars denote mean ± SD with n = 3. SDs for VOC and ISC were fixed at 4% and 6% of the mean, respectively; these were propagated to power using P = VOCISC/2, yielding a 7.2% relative SD for both power and power density (active area = 16 cm^2^). Peak performance occurs at 0.03–0.05 wt% MWCNT.

Across the 0.01–0.03 wt% MWCNT loading range, the dielectric permittivity (ε′) increases while electrical conductivity (σ) remains nearly constant at low frequencies, indicating enhanced charge storage without excessive conductive loss. In the 0.03–0.05 wt% range, interfacial (Maxwell–Wagner–Sillars) polarization is maximized while dielectric loss remains controlled, yielding the highest device outputs: P = 129.6 ± 9.33 µW (81.0 ± 5.83 mW m^-2^) at 0.03 wt% and P = 145.35 ± 10.47 µW (90.8 ± 6.54 mW m^−2^) at 0.05 wt% (mean ± SD; n = 3). Although the mean output at 0.05 wt% is ∼12% higher, the overlapping uncertainties indicate that this difference is not statistically significant; therefore, a co-optimal concentration window of 0.03–0.05 wt% is identified rather than a single sharp optimum. At higher loading (0.10 wt%), only marginal gains in ε′ are observed, while ε″ and σ increase due to agglomeration-induced tunneling and near-percolation pathways, resulting in degraded performance. Overall, these results demonstrate that uniform MWCNT dispersion within the 0.03–0.05 wt% range provides an optimal balance between strong interfacial polarization and limited dielectric loss.

In conclusion, the experimental findings confirm that the controlled incorporation of MWCNTs into the PS matrix markedly enhances the performance of TENG devices. The observed improvements in open-circuit voltage, short-circuit current, output power, and power density arise from synergistic effects such as increased dielectric constant, strengthened interfacial polarization, and improved charge transport. However, these enhancements critically depend on maintaining uniform nanotube dispersion and preventing agglomeration. Excessive filler loading may lead to inferred structural irregularities and increased conductive losses, highlighting the importance of careful nanofiller optimization to maintain TENG efficiency.

## 4 Limitations and future perspectives

While the present study establishes a clear structure–property–performance relationship for polysiloxane/MWCNT-based TENGs, several limitations should be acknowledged, which also point to important directions for future research. First, the identification of a co-optimal MWCNT concentration window (0.03–0.05 wt%) is based on overlapping uncertainties in the measured triboelectric outputs (Voc, Isc, and power density). Although the mean values indicate a modest performance increase at 0.05 wt%, formal statistical separation between the 0.03 wt% and 0.05 wt% samples are not supported within the current experimental uncertainty and sample size (n = 3). Therefore, the reported range should be interpreted as a practical design window rather than a sharply defined optimum. Future studies employing larger numbers of independently fabricated devices and finer concentration intervals would enable more rigorous statistical discrimination.

Second, the mechanistic interpretation of performance degradation at higher MWCNT loadings (≥0.10 wt%) is primarily inferred from dielectric trends, including increased dielectric loss and conductivity consistent with near-percolation behavior. Direct nanoscale visualization of filler dispersion and agglomeration, such as cross-sectional SEM or TEM analysis, was not performed in the present work. Incorporating such structural characterization in future investigations would provide direct physical validation of the proposed mechanisms and further strengthen structure–performance correlations.

Third, the current evaluation focuses on short-term electrical output under controlled laboratory conditions. Long-term operational stability, mechanical fatigue under repeated deformation, and environmental robustness against humidity and temperature fluctuations were not systematically addressed. These factors are essential for assessing practical deployment in wearable and flexible energy-harvesting systems and represent key targets for future studies.

Finally, future work may explore process-level optimization and material generalization, including the influence of film thickness, electrode configuration, and alternative counter-triboelectric materials, as well as the extension of the low-filler strategy to other elastomeric matrices. Such efforts could further enhance performance scalability and accelerate the translation of optimized nanocomposite TENGs toward real-world applications.

## 5 Conclusion

The experimental investigation demonstrates that incorporating functionalized multi-walled carbon nanotubes (f-MWCNTs) into the polysiloxane (PS) matrix effectively enhances the triboelectric nanogenerator (TENG) performance through controlled modulation of dielectric and interfacial properties. SEM analysis confirmed uniform dispersion of f-MWCNTs at low concentrations (≤0.05 wt%), supporting optimal interfacial contact and charge transport pathways. For higher concentrations, aggregation is inferred from dielectric trends rather than directly observed. The output characteristics of the fabricated devices revealed a pronounced dependence on nanofiller concentration. Both the open-circuit voltage (Voc) and short-circuit current (Isc) increased significantly with f-MWCNT loading up to 0.03–0.05 wt%. Beyond this range, a decline in performance was observed, which may be attributed to inferred agglomeration and the possible onset of near-percolation effects, as suggested by the dielectric and conductivity trends.

Dielectric measurements indicated that within the 0.01–0.03 wt% range, the real part of permittivity (ε′) increased while conductivity (σ) remained relatively stable at low frequencies, signifying efficient charge storage with minimal loss. Around 0.03–0.05 wt%, interfacial (Maxwell–Wagner–Sillars) polarization reached its maximum, while conductive losses remained moderate, resulting in the highest device outputs: P = 129.6 ± 9.33 µW (81.0 ± 5.83 mW m^−2^) at 0.03 wt% and P = 145.35 ± 10.47 µW (90.8 ± 6.54 mW m^−2^) at 0.05 wt% (mean ± SD; n = 3). Despite the ∼12% difference, overlapping uncertainties suggest a co-optimal range between 0.03 and 0.05 wt% MWCNT. At higher concentrations (0.10 wt%), ε′ increased marginally, but ε″ and σ rose sharply due to tunneling and conductive network formation, reducing the triboelectric output.

Overall, the results confirm that the enhancement of TENG performance arises from the synergistic effects of increased dielectric constant, improved interfacial polarization, and more efficient charge transport, all of which depend on maintaining uniform nanotube dispersion. Excessive nanofiller loading may induce inferred agglomeration and conductive losses, emphasizing the importance of careful control of filler content. Thus, maintaining MWCNT content within the narrow window of 0.03–0.05 wt% ensures a balanced combination of high polarization, low loss, and superior energy output, providing a reliable strategy for designing next-generation high-performance triboelectric nanogenerators.

## Data Availability

The original contributions presented in the study are included in the article; further inquiries can be directed to the corresponding author.
